# A Practical Treatise on the Diseases of the Hair and Scalp

**Published:** 1894-12

**Authors:** 


### BOOK NOTICES AND REVIEWS.

A Practical Treatise on the Diseases of the Hair and Scalp. By George Thomas Jackson, M. D. New, revised and enlarged edition. New York: E. B. Treat, 5 Cooper Union. 1894. Price, $2.75.

This book is, like some others of recent issue, a miniature encyclopædia of the subject of the hair and scalp. The present work is a new edition, the first having been issued in 1887.

The author was induced to write this volume because of the urgent need of some complete treatise on the hair and scalp that was scientific in character.

For this purpose he has scanned an immense mass of literature for his materials. His industry in this search is demonstrated by turning to the end of the book, where we find a catalogue of journals and books consulted that occupies forty-four pages of fine type.

The book opens with a chapter on the anatomy of the hair, a second chapter upon its physiology, and a third upon the hygiene of the hair.

These chapters constitute Part First.

Part Second concerns the essential diseases of the hair, Part Third the parasitic diseases, and Part Fourth diseases of the hair secondary to diseases of the skin.

The index is quite full, as an index ought always to be to make a book of any value.

The hygiene of the hair and scalp consists of the proper use of the shampoo, in brushing and combing, in arranging the hair, in exposure of the hair to air and light, in cutting and shaving it, and in the use of pomades.

For shampooing, the proper manner is to make a lather on the head with some good soap—several of which are mentioned—and then give the scalp a vigorous rubbing with the fingers or stiff, long-bristled brush.

The author also recommends Borax instead of soap, or a mixture of yolk of eggs and lime water. After thorough washing and drying, rub in some oily substance like sweet almond oil or vaseline.

The drying of the hair, especially of women, should be carefully done before a fire or in the direct sunlight.

Careful directions for the proper oiling of the scalp are given. The same with brushing and combing.

He objects to the whole procedure of curling irons, curl papers, singeing, squeezing between hot irons, and scorching.

In the cutting of men's hair he denounces the prevailing custom of shingling, as it tears the hair and roughens it.

The author objects to shaving, as depriving the throat and lungs of suitable protection.

He does not like pomades. Their use is uncalled for. They are dirty and soon become rancid, and so emit a foul odor.

As the hair sympathizes with the general health of the body, keeping the latter in good condition may postpone for years the advent of baldness.

